# Effects of Decabrominated Diphenyl Ether (PBDE-209) in Regulation of Growth and Apoptosis of Breast, Ovarian, and Cervical Cancer Cells

**DOI:** 10.1289/ehp.1104051

**Published:** 2012-01-06

**Authors:** Zhi-Hua Li, Xiao-Yan Liu, Na Wang, Jing-Si Chen, Yan-Hong Chen, Jin-Tao Huang, Chun-Hong Su, Fukang Xie, Bin Yu, Dun-Jin Chen

**Affiliations:** 1 Department of Obstetrics and Gynecology, Third Affiliated Hospital of Guangzhou Medical College, Key Laboratory for Major Obstetric Diseases of Guangdong Province, Guangzhou, China; 2 State Key Laboratory of Biocontrol, School of Life Sciences, Sun Yat-sen (Zhongshan) University (East Campus), Guangzhou, China; 3 Department of Histology and Embryology, Medical School of Sun Yet-Sen University, Guangzhou, China

**Keywords:** cell proliferation, ERK1/2, female reproductive cancer, PBDE-209, PKCα

## Abstract

Background: Polybrominated diphenyl ethers (PBDEs), commonly used in building materials, electronics, plastics, polyurethane foams, and textiles, are health hazards found in the environment.

Objective: In this study we investigated the effects of PBDE-209, a deca-PBDE, on the regulation of growth and apoptosis of breast, ovarian, and cervical cancer cells as well as the underlying protein alterations.

Methods: We used MCF-7 and MCF-7/ADR (multidrug-resistant MCF-7) breast cancer cell lines, the HeLa cervical cancer cell line, the OVCAR-3 ovarian cancer cell line, and the normal CHO (Chinese hamster ovary) cell line to assess the effects of PBDE-209 using cell viability, immunofluorescence, and flow cytometric assays. Western blot assays were used to detect changes in protein expression. To assess the effects of PBDE-209 on apoptosis, we used the protein kinase Cα (PKCα) inhibitor Gö 6976, the extracellular signal-regulated kinase (ERK) inhibitor PD98059, and tamoxifen.

Results: Our data indicate that PBDE-209 increased viability and proliferation of the tumor cell lines and in CHO cells in a dose- and time-dependent manner. PBDE-209 also altered cell cycle distribution by inducing the S phase or G_2_/M phase. Furthermore, PBDE-209 partially suppressed tamoxifen-induced cell apoptosis in the breast cancer cell lines (MCF-7 and MCF-7/ADR) but suppressed Gö 6976- and PD98059-induced apoptosis in all cell lines. At the molecular level, PBDE-209 enhanced PKCα and ERK1/2 phosphorylation in the cell lines.

Conclusions: Our data demonstrate that PBDE-209 is able to promote proliferation of various cancer cells from the female reproductive system and normal ovarian CHO cells. Furthermore, it reduced tamoxifen, PKCα, and ERK inhibition-induced apoptosis. Finally, PBDE-209 up-regulated phosphorylation of PKCα and ERK1/2 proteins in tumor cells and in CHO cells.

Polybrominated diphenyl ethers (PBDEs) are flame retardants commonly used in an array of products, including construction materials, electronics, furnishings, plastics, polyurethane foams, and textiles (Brown et al. 2004). PBDEs are found in various environmental media (Bi et al. 2006; Guan et al. 2009; Thomsen et al. 2010) and are health hazards. They are toxic, persistent, and bioaccumulative and could induce endocrine-disrupting activity (Darnerud et al. 2001; Hamers et al. 2006, 2008; Richardson et al. 2008). Thus, the Stockholm Convention approved the elimination of their industrial production and use. Nevertheless, commercial PBDE mixtures are used without regulation in most Asian countries (Tan et al. 2007). Deca-PBDE is the only PBDE technical mixture currently produced in large quantities worldwide, a major component of which is PBDE congener 209 (La Guardia et al. 2006). Evidence indicates that PBDEs can cause neurobehavioral deficits and possibly cause cancer in experimental animals (McDonald 2002).

Molecularly, PBDEs elicit thyroxine-like and estrogen-like activity *in vitro* (Meerts et al. 2000). Barber et al. (2006) showed that low doses of PBDE (10^–12^ to 10^–9^ M) induce growth kinetics and micronucleus formation in MCF-7 breast cancer cells. Llabjani et al. (2010, 2011) and Ukpebor et al. (2011) also found that low doses of PBDE induce MCF-7 cell proliferation. Mercado-Feliciano and Bigsby (2008) showed that the PBDE mixture DE-71 increases MCF-7 cell proliferation, which was prevented by antiestrogen treatment. PBDEs affect both male and female reproductive systems *in vivo* (Ceccatelli et al. 2006; Kuriyama et al. 2005; Lilienthal et al. 2006; Stoker et al. 2004; Talsness et al. 2005; Tseng et al. 2006). Metabolically, PBDE congeners PBDE-47, PBDE-85, and PBDE-99 are selectively taken up and retained in the liver, adrenal cortex, and ovaries after PBDE exposure in adult C57BL mice (Darnerud and Risberg 2006). Talsness et al. (2008) demonstrated that exposure to low concentrations of PBDE-47 *in utero* and during lactation decrease the offspring’s ovarian weight and size of tertiary follicles. These studies indicate that PBDEs may significantly affect the reproductive system and be responsible for increasing cancer incidence in the mammary glands, uterus, and ovary. Therefore, in the present study we investigated the effects of PBDEs on breast, cervical, and ovarian cancer cell lines using normal Chinese hamster ovary (CHO) cells as the control. We also investigated the effects of PBDE-209 on regulation of tamoxifen-induced apoptosis in breast cancer cells because PBDE has been shown to disrupt hormones, including estrogen. We then examined the underlying molecular mechanisms by which PBDEs induce protein alterations.

## Materials and Methods

*Cell lines and culture.* The MCF-7 human breast cancer cell line, the multidrug-resistant MCF-7 cell line MCF-7/ADR, and OVCAR-3 human ovarian cancer cell line were a generous gift from W. Huang (Sun Yat-Sen University, Guangzhou, China). We obtained the HeLa human cervical cancer cell line and CHO (Chinese hamster ovary) cell line from the Experimental Animal Center at Sun Yat-Sen University.

MCF-7 and MCF-7/ADR cells were maintained in RPMI-1640 medium, OVCAR-3 and HeLa cells were cultured in HEPES-buffered Dulbecco’s modified Eagle’s medium (H-DMEM), and CHO cells were maintained in DMEM-F12 medium. All media were supplemented with 10% fetal bovine serum (FBS), 100 U/mL penicillin, and 100 μg/mL streptomycin (all from Gibco BRL, Grand Island, NY, USA). Cells were cultured at 37°C in a humidified atmosphere of 95% air and 5% CO_2_. Third- or fourth-passage cells were used for our experiments. MCF-7 and MCF-7/ADR cells were grown in phenol red–free RPMI-1640 supplemented with 10% DCC-FBS (dextran-coated charcoal–stripped FBS; Sigma Chemical Co., St. Louis, MO, USA) according to Migliaccio et al. (1993). After 24 hr culture, cells were starved overnight with phenol red–free RPMI-1640 containing 1% DCC-FBS (for breast cancer cells), H-DMEM (for OVCAR-3 and HeLa cells), or DMEM-F12 with 1% FBS (for CHO cells). Cells were treated with PBDE-209, tamoxifen, the protein kinase Cα (PKCα) inhibitor Gö 6976, or the extracellular signal-regulated kinase (ERK) inhibitor PD98059 (all from Sigma) added into the culture medium as described for specific experiments.

*Cell viability by MTT (methylthiazolyldiphenyl tetrazolium bromide) assay.* We used the MTT assay to assess cell viability (Mosmann 1983). Cells were seeded in 96-well plates at approximately 10^3^–10^4^ cells/well in 200 μL medium for 24 hr, followed by starvation for another 24 hr. The cells were then divided into four groups in triplicate: *a*) blank control; *b*) dimethyl sulfoxide (DMSO) vehicle control; *c*) PBDE-209 at a final PBDE-209 concentration of 5, 15, 25, 50, or 100 nM; and *d*) 10% FBS positive control (cells cultured only with medium plus 10% FBS). The percent cell viability was calculated as (A490 of treated cells – A490 of blank control) ÷ (A490 of negative control – A490 of blank control) × 100%, as described previously (Shen et al. 1995).

*Cell proliferation by immunofluorescent staining of Ki67.* Cells were grown and treated as above and then subjected to immunofluorescent staining of Ki67, a marker for cell proliferation. Briefly, the cells were fixed in 4% paraformaldehyde, treated with 0.01% Triton X-100 for 30 min, incubated with 5% bovine serum albumin in phosphate-buffered saline (PBS) for 30 min and then incubated with the Ki67 antibody (1:200; Lab Vision, Fremont, CA, USA) overnight at 4°C. The next day, cells were washed with PBS and incubated with Cy3-labeled goat anti-rabbit secondary antibody (Sigma) at room temperature for 2 hr and with 0.001% DAPI (4´,6-diamidino-2-phenylindole)/PBS for 15 min. The cells were submerged, resuspended in glycerol/PBS, reviewed, and scored using an inverted fluorescence microscope (Leica, Wetzlar, Germany). Images were recorded and merged using Adobe Photoshop CS software (Adobe Systems Inc., San Jose, CA, USA).

*Flow cytometry assay.* We measured cell cycle distribution and apoptosis using propidium iodide staining and a flow cytometer (model FC500; Beckman-Coulter, Brea, CA, USA) as described previously by Mandil et al. (2001). Briefly, tumor cells were grown and treated with 0–100 nM PBDE-209 for 72 hr. Breast cancer cells (MCF-7 and MCF-7/ADR) were also co-treated with 5 μM tamoxifen in the presence of 50 or 100 nM PBDE-209 for 72 hr. In addition, the cells were pretreated independently with 0.1 μM Gö 6976 or 20 μM PD98059 for 30 min before treatment with 0, 25, 50, or 100 nM PBDE-209 for 72 hr. Each experiment was repeated in triplicate.

*Protein extraction and Western blotting.* The cells were lysed according to a protocol described previously (Li et al. 1999). We obtained rabbit polyclonal antibodies against phosphorylated PKCα, phosphorylated ERK, and β-actin from Cell Signaling Technology (Beverly, MA, USA); horseradish peroxidase–conjugated rabbit or secondary antibodies from QED Biovision, Inc. (Hercules, CA, USA); and enhanced chemiluminescence detection equipment from GE Healthcare (Chalfont St. Giles, UK).

*Statistical analysis.* Statistical analysis was performed with the SPSS software (version 11.5; SPSS, Chicago, IL, USA). Data are expressed as mean ± SD. We used independent-samples *t*-test and analyses of variance to assess the statistical significance of the samples. The difference is considered statistically significant at *p* < 0.05.

## Results

**Figure 1:**
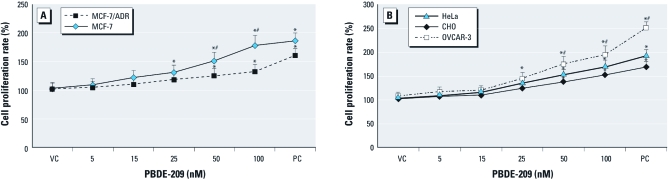
Cell viability in MCF‑7 and MCF‑7/ADR cells (*A*) and in OVCAR‑3, HeLa, and CHO cells (*B*) determined by MTT assay. Cells were grown, starved overnight without serum, treated with different doses of PBDE‑209 for 72 hr in medium containing 1% serum, and then subjected to MTT assay. Cells treated with 0.1% DMSO were used as the vehicle control (VC), and 10% FBS-treated cells were used as the positive control (PC). The experiments were performed three times. Values shown are mean ± SD. *p < 0.05 versus VC. ^#^*p* < 0.05 versus MCF‑7/ADR cells (*A*) or versus CHO cells (*B*) in the same treatment group.

*PBDE-209 induced phenotypic changes.* To assess the effects of PBDEs on different tumor cell lines, we treated cells with up to 100 nM PBDE-209 for 72 hr and evaluated if there were changes in their morphology. We found that, with increasing PBDE-209 dose, cells grew more rapidly and were closer to each other; thus the cell gaps were decreased compared with control cells, which was more obvious in MCF-7 cells than in MCF-7/ADR cells and more obvious in OVCAR-3 cells than in CHO cells. The cell viability assay showed that the absorption reading of PBDE-209–treated cells increased gradually as the PBDE-209 dose increased (Figure 1). For example, MCF-7 cells with 25 nM PBDE-209 treatment had a proliferative rate 1.22 times higher than that of control cells, and the proliferative rate in MCF-7 cells treated with 100 nM PBDE-209 was 1.85 times higher than that of control cells. In contrast, MCF-7/ADR cells were less sensitive to PBDE-209 than were MCF-7 cells: the proliferative rate of cells treated with 50 nM PBDE-209 was only 1.25 times higher than that of control cells and 1.32 times higher in cells treated with 100 nM PBDE-209 (*p* < 0.05; Figure 1). Moreover, OVCAR-3 cells responded to PBDE-209 more strongly than did normal ovarian CHO cells: Increasing the PBDE-209 dose to 100 nM led to a proliferative rate of 1.86 in OVCAR-3 cells, compared with about 1.50 in CHO cells (*p* < 0.05). However, the response of HeLa cells to PBDE-209 was similar to that of CHO cells. These data indicate that response of these tumor cells to PBDE-209 treatment was dose and cell line dependent.

**Figure 2:**
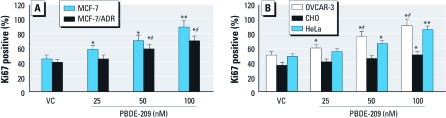
Detection of Ki67 expression in MCF‑7 and MCF‑7/ADR cells (*A*) and in OVCAR‑3, HeLa, and CHO cells (*B*) by immunofluorescence. Cells were grown, starved overnight without serum, treated with different doses of PBDE‑209 for 72 hr in medium containing 1% serum, and then subjected to immunofluorescence analysis of Ki67 expression. Cells treated with 0.1% DMSO were used as vehicle control (VC). The experiments were performed three times. Ki67 positivity is presented as the ratio of Ki67-positive cells to the number of all cells in that cell line; data shown are mean ± SD. *p < 0.05 versus VC. **p < 0.01 versus VC. ^#^*p* < 0.05 versus MCF‑7/ADR cells (*A*) or versus CHO cells (*B*) in the same treatment group.

We also determined the effects of PBDE-209 on induction of tumor cell proliferation using Ki67 immunostaining. The data showed that after PBDE-209 treatment for 72 hr, the number of Ki67-positive cells increased and the intensity of Ki67 expression was stronger compared with control cells without PBDE-209 treatment (Figure 2).

**Figure 3:**
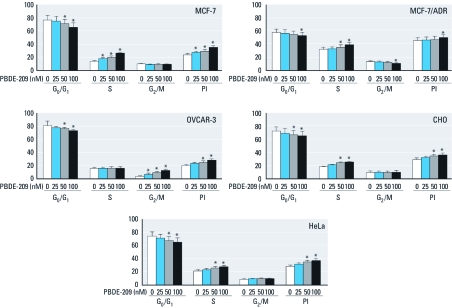
Cell cycle profile determined by flow cytometry. Cells treated with 0.1% DMSO were used as vehicle control (VC; 0 nM PBDE‑209). The experiments were performed three times. Data shown are the percentage of cells at that phase of the cell cycle (mean ± SD). *p < 0.05 versus 0 nM PBDE‑209.

*PBDE-209 induced cell cycle alterations.* Khan and Dipple (2000) showed that anti-benzo[*a*]pyrene antibody could induce evasion of G_1_ arrest to promote tumorigenicity, so we performed flow cytometry to detect the effects of PBDE-209 on cell cycle alteration in these tumor cell lines. To our surprise, PBDE-209 decreased the percentage of cells in the G_0_/G_1_ phase after 72 hr treatment but elevated the percentage of cells in the S phase, except for OVCAR-3 cells, which showed an increase in the G_2_/M phase (Figure 3). Data indicate that PBDE-209 increased the proliferation index [PI; PI = (S + G_2_M) ÷ (G_0/_G_1_ + G_2_M)] after 72 hr of treatment (*p* < 0.05; Figure 3). The percentage of G_0_/G_1_ phase cells in the MCF-7/ADR cell was only 50–60%, and the percentage of S phase cells reached > 37% (Figure 3). Although the PI of the PBDE-209 test group was higher than that of control cells, the difference was significant only in cells treated with 100 nM PBDE-209 (Figure 3).

*PBDE-209 partially suppressed tamoxifen-induced apoptosis in breast cancer cells.* We examined the effects of PBDE-209 on regulation of tamoxifen-induced apoptosis in breast cancer cells because PBDE has been shown to disrupt hormones, including estrogen. We also examined expression of estrogen receptor α (ERα) and ERβ in breast cancer cells using immunofluorescence, Western blot, and reverse-transcriptase polymerase chain reaction (RT-PCR), as described in Supplemental Material (http://dx.doi.org/10.1289/ehp.1104051). ERα and ERβ were expressed in MCF-7 cells but not in MCF-7/ADR cells (see Supplemental Material, Figures 1 and 2), which confirmed data from a previous study (Monje and Boland 2002).

**Figure 4:**
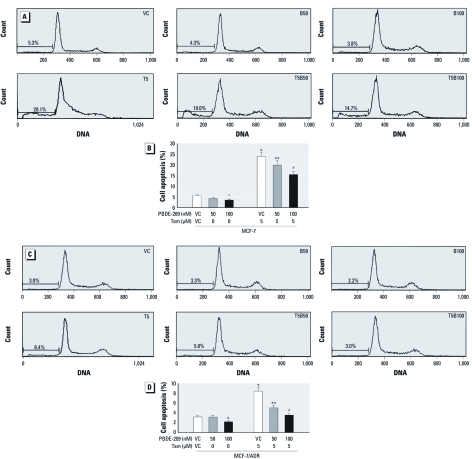
Effect of PBDE‑209 on regulation of tamoxifen-induced apoptosis in MCF‑7 (*A,B*) and MCF‑7/ADR (*C,D*) breast cancer cells. Cells were grown, starved overnight without serum, treated with different doses of PBDE‑209 (PBDE) with or without 5 μM tamoxifen for 72 hr in medium containing 1% serum, and then subjected to flow cytometry. Cells treated with 0.1% DMSO were used as vehicle control (VC). Abbreviations: B50, 50 nM PBDE-209; B100, 100 nM PBDE-209; T5, 5 μM tamoxifen; T5B50, 5 μM tamoxifen + 50 nM PBDE-209; T5B100, 5 μM tamoxifen + 100 nM PBDE-209. Histograms (*A,C*) present flow cytometric data, and the bar charts (*B,D*) present percentages of apoptosis. The experiments were performed three times, and the data shown in *B* and *D* are mean ± SD of three values. *p < 0.05 versus VC. **p < 0.05 versus tamoxifen alone. ^#^*p* < 0.01 versus tamoxifen alone.

We also assessed the apoptosis ratio by flow cytometry in MCF-7 cells and MCF-7/ADR cells after treatment with 5 μM tamoxifen in the presence of 50 nM or 100 nM PBDE-209. Figure 4 shows that the apoptosis rate in breast cancer cells exposed to 100 nM PBDE-209 alone was lower than that in control cells. Moreover, PBDE-209 was able to reduce tamoxifen-induced apoptosis in both MCF-7 cells and MCF-7/ADR cells. In MCF-7 cells the apoptosis rate decreased from 23.8% in the tamoxifen group to 19.7% in the tamoxifen plus 50 nM PBDE-209 group and to 15.4% in the tamoxifen plus 100 nM PBDE-209 group (Figure 4A). In MCF-7/ADR cells the apoptosis rate decreased from 8.4% to 5.0% and to 3.5%, respectively, in the three groups (Figure 4B). It is clear that treatment with 100 nM PBDE-209 suppressed tamoxifen-induced apoptosis in breast cancer cells (*p* < 0.05).

**Figure 5:**
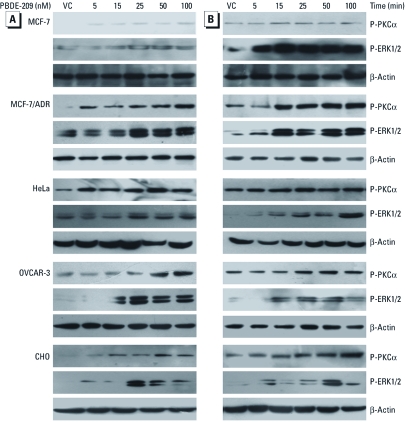
Evaluation of PKCα and ERK1/2 phosphorylation (P) in cells shown by Western blotting. Cells were grown, starved for 24 hr without serum, and treated with different doses of PBDE‑209 (up to 100 nM) for 15 min in medium containing 1% serum (*A*), or treated with 50 nM PBDE‑209 for up to 120 min (*B*). Cells were then subjected to Western blotting. Cells treated with 0.1% DMSO were used as vehicle control (VC). The experiments were repeated twice, with similar data for both experiments. β‑Actin was used as a loading control.

*PBDE-209 induced PKC*α *and ERK1/2 phosphorylation.* Our data clearly demonstrate that PBDE-209 leads to changes in cell proliferation, cell cycle, and apoptosis. We therefore investigated the underlying mechanism by which PBDE-209 alters protein expression. Activation of PKCα and ERK proteins plays a role in cell proliferation. In dose-dependent experiments, we observed that after 15 min of treatment with PBDE-209, PKCα phosphorylation was increased in all five cell lines and peaked at a dose of 50 nM or 100 nM, whereas ERK phosphorylation reached a maximum level at 25 nM but remained high up to 100 nM PBDE-209 (Figure 5A). For time-dependent experiments in which cells were treated with 50 nM PBDE-209 for up to 120 min (Figure 5B), PKCα phosphorylation was apparent after 15 min of treatment and reached a maximum at 60 or 120 min, except for MCF-7 cells, which reached a maximum at 15 min. Furthermore, PBDE-209-induced ERK1/2 phosphorylation peaked at 15 min in MCF-7, MCF-7/ADR, and OVCAR-3 cells and was maintained up to 120 min in MCF-7 and MCF-7/ADR cells and up to 60 min in OVCAR-3 cells. In HeLa cells, ERK was activated after 15 min exposure to PBDE-209 and then gradually rose to maximal levels at 120 min, whereas in CHO cells, ERK phosphorylation reached a peak after 1 hr of treatment (Figure 5B).

**Figure 6:**
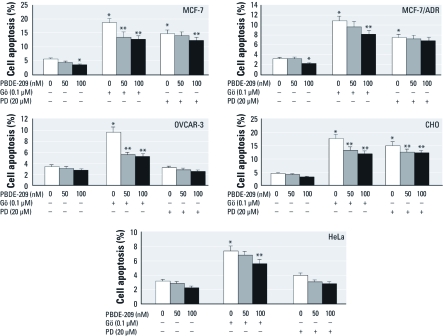
Effects of PBDE‑209 on regulation of apoptosis induced by the PKCα inhibitor Gö 6976 or the ERK inhibitor PD98059. Cells were pretreated with 1 μM Gö 6976 (Gö) or 20 μM PD98059 (PD) for 30 min and then treated with 0, 50, or 100 nM PBDE‑209 for 72 hr. Cells treated with 0.1% DMSO were used as the vehicle control (VC; 0 nM PBDE‑209). Data shown are mean ± SD of three experiments. *p < 0.05 versus VC. **p < 0.05 versus Gö 6976 or PD98059 treatment alone.

*Effects of PBDE-209 on regulation of Gö 6976 and PD98059-induced apoptosis.* We investigated the effects of PBDE-209 on regulation of the apoptosis induced by the PKCα inhibitor Gö 6976 and the ERK inhibitor PD98059. Figure 6 shows that both Gö 6976 (1 μM) and PD98059 (20 μM) were able to induce apoptosis, especially in MCF-7 and CHO cells (*p* < 0.05). Specifically, the apoptosis rate induced by Gö 6976 was higher than that induced by PD98059. In contrast, the apoptosis rate was reduced by PBDE-209 treatment in MCF-7 and MCF-7/ADR cells compared with vehicle controls (0 nM PBDE-209; *p* < 0.05), but it was not reduced in HeLa, CHO, or OVCAR-3 cells. Moreover, in all cells, 100 nM PBDE-209 reduced Gö 6976-induced apoptosis more than did 50 nM PBDE-209 (Figure 6). However, only 100 nM PBDE-209 reduced PD98059-induced apoptosis in MCF-7 cells and CHO cells (Figure 6).

## Discussion

PBDEs are structurally similar to other environmental contaminants, such as polychlorinated biphenyls and organochlorines, which are potential carcinogens according to reports from the International Agency for Research on Cancer (1978, 1991). Thus, PBDEs may have similar properties, such as environmental persistence and lipophilicity, that might be able to induce intracellular and organism toxicity (Birnbaum and Staskal 2004; Helleday et al. 1999). However, investigation into the toxicity and carcinogenicity of PBDE-209 in humans remains surprisingly limited and uncertain. In the present study, we demonstrate that PBDE-209 induces proliferation of breast, cervical, and ovarian cancer cells and normal ovarian CHO cells in a dose-dependent manner. However, our data contradict data from Hu et al. (2007), which showed that PBDE-209 inhibited hepatoma cell viability in a time- and concentration-dependent fashion at doses between 10 and 100 μM. The difference between the two studies may be because the doses they used were 100–1,000 times higher than those we used, and doses that high may not be achievable in humans through environmental exposure. Moreover, in our study PBDE-209 antagonized Gö 6976- and PD98059-induced apoptosis in cancer cells and partially antagonized tamoxifen-induced breast cancer cell apoptosis. However, it remains unknown whether this is related to the fact that some advanced breast cancers initially respond well to tamoxifen but eventually become resistant to it. Whether PBDE-209 affects cervical and ovarian cancer treatment needs to be confirmed using clinical data, for example, data from areas where PBDE is highly used [e.g., Zhejiang province of China, where morbidity from lung, kidney, and liver cancer is higher (Zhao et al. 2009)].

Furthermore, PBDEs can also elicit thyroidogenic-like and estrogen-like activity *in vitro* (Meerts et al. 2000). Thus, we chose ER-positive breast cancer cells (MCF-7) and ER-negative breast cancer cells (MCF-7/ADR) (Kalantzi et al. 2004) to assess the effect of PBDE-209 in this study. We used phenol red–free growth medium and DCC-FBS to eliminate the effects of estrogen in the cells and found that 100 nM PBDE-209 could induce cell proliferation 1.77-fold greater in MCF-7 cells than in MCF-7/ADR cells, but estrogen could not be detected in the culture medium of either of these breast cancer cells after 100 nM PBDE-209 exposure for 7 days (data not shown). Data from the present study indicate that PBDE-209 has multiple toxic functions in these cells, perhaps not through the ER pathway, because PBDE-209 can induce cell proliferation in both ER-positive and ER-negative cells.

PKCα is one of 12 members of the PKC family, which are serine/threonine kinases involved in many important cellular functions, such as cell proliferation, migration, differentiation, and apoptosis (Clemens et al. 1992; Hug and Sarre 1993; Tanaka et al. 2003). Mitogen-activated protein kinases include four different types of proteins, that is, ERK1/2, c-jun N-terminal kinase/stress-activated protein kinase (JNK/SAPK), big MAP kinase 1 (BMK1 or ERK5), and the p38 group of protein kinases (Zarubin and Han 2005), which are serine/threonine protein kinases that are activated by diverse stimuli, including cytokines, growth factors, neurotransmitters, hormones, cellular stress, or cell adherence. Activation of PKCα/ERK proteins has been reported to promote cell proliferation (Fujii et al. 2005) but inhibit apoptosis (Tian et al. 2009). Data from the present study show that PBDE-209 induced phosphorylation of PKCα and ERK1/2 proteins in both MCF-7 and MCF-7/ADR cells. Our finding is similar to that of Mercado-Feliciano and Bigsby (2008), who showed that PBDE-209 induced cell proliferation in MCF-7 cells, but induction was blocked by antiestrogen treatment.

Previous studies revealed that PBDE bioaccumulates in the ovary and changes its structure (Darnerud and Risberg 2006; Talsness et al. 2008). However, the role of PBDE in the ovary remains unknown. In the present study, we showed that PBDE-209 promotes growth of CHO (normal ovary) cells and OVCAR-3 ovarian cancer cells. PBDE-induced CHO cell proliferation was mediated through induction of the S phase of the cell cycle, whereas PBDE-increased OVCAR-3 cell growth was mediated through an elevation of the G2/M phase. However, the differential effect of PBDE on normal and cancerous ovarian cells remains to be determined.

## Conclusion

PBDE-209 (up to 100 nM) induced not only proliferative effects but also antiapoptotic effects in different cancer cell lines and activated PKCα and ERK1/2 phosphorylation. It also antagonized apoptosis induced by the PKCα inhibitor Gö 6976 and the ERK inhibitor PD98059 in these cells and partially antagonized tamoxifen-induced breast cancer cell apoptosis. Further study is needed to determine whether PBDE exposure is associated with cancer development in the female reproductive system, but avoiding exposure to PBDE-209 is a reasonable precaution.

## Supplemental Material

(152 KB) PDF
