# Supplementary material for: Effects of Decabrominated Diphenyl Ether (PBDE-209) in Regulation of Growth and Apoptosis of Breast, Ovarian, and Cervical Cancer Cells
Source: Environ Health Perspect. 2012 Jan 6;120(4):541–6. doi: 10.1289/ehp.1104051 (PMC3339458; doi:10.1289/ehp.1104051)
Supplement: (152 KB) PDF [file ehp.1104051.s001.pdf]

Supplemental Material

Effects of Decabrominated Diphenyl Ether (PBDE-209) in Regulation of Growth and Apoptosis of Breast, Ovarian, and Cervical Cancer Cells

Zhi-Hua Li [1], Xiao-Yan Liu [1] , Na Wang [2], Jing-Si Chen [1], Yan-Hong Chen[1], Jin-Tao Huang [3], Chun-Hong Su [1], Fu-kang Xie[3], Bin Yu[1] and Dun-Jin Chen [1]

[1]Department of Obstetrics and Gynecology, the Third Affiliated Hospital of Guangzhou Medical College, Guangzhou 510150, China.

[2]State Key Laboratory of Biocontrol, School of Life Sciences, Sun Yat-sen (Zhongshan) University (East Campus), Bei San Road, Guangzhou 510275, China

[3]Department of Histology and Embryology, Medical School of Sun Yet-Sen University, Guangzhou 510080, China.

Address correspondence to D-J. Chen , Department of Obstetrics and Gynecology, the Third Affiliated Hospital of Guangzhou Medical College, 63 Duobao Road, Guangzhou, Guangdong 510150, China. Tel.: +86-20-81292211; Fax: +86-20-81292127; E-mail: chendunjin@hotmail.com.cn

Figures content: 2 figures

Materials and Methods

ERα expression detected by immunofluorescent staining in breast cancer cells **:** Breast cancer cells were grown in RPMI-1640 medium with 10% fetal bovine serum (FBS), 100 U/mL penicillin, and 100 μg/mL streptomycin (all from Gibco BRL, Grand Island, NY, USA) for 72h, and then subjected to immunofluorescent staining of *ERα*. Briefly, the cells were fixed in 4% paraformaldehyde, treated with 0.01% Triton X-100 for 30 min, incubated with 5% bovine serum albumin in phosphate-buffere saline (PBS) for 30 min and then incubated with the *ERα* antibody (1:200; Lab Vision, Fremont, CA, USA) overnight at 4° C. The next day, cells were washed with PBS and incubated with Cy3-labeled goat anti-rabbit secondary antibody (Sigma) at room temperature for 2 h and with 0.001% DAPI (4′,6-diamidino-2-phenylindole)/PBS for 15 min. The cells were submerged, resuspended in glycerol/PBS, reviewed, and scored using an inverted fluorescence microscope (Leica, Wetzlar, Germany). Images were recorded and merged using Adobe Photoshop CS software (Adobe Systems Inc., San Jose, CA, USA).

ERα expression detected by Western Blot in breast cancer cells: Breast cancer cells were grown in RPMI-1640 medium with 10% fetal bovine serum (FBS), 100 U/mL penicillin, and 100 μg/mL streptomycin (all from Gibco BRL, Grand Island, NY, USA) for 72 h, following the protein extraction and Western Blot for rabbit polyclonal antibodies ERα, β-actin as the control.

Reverse transcription (RT)-PCR analysis: MCF-7 and MCF-7/ADR cells were grown in RPMI-1640 medium with 10% fetal bovine serum (FBS), 100 U/mL penicillin, and 100 μg/mL streptomycin, and total RNA was isolated using TRIzol reagent (Invitrogen) and treated with DNase I (Takara) to remove genomic DNA. The first strand of complementary DNA (cDNA) was synthesized from 1 μg of total RNA using reverse transcriptase kit (Takara) and oligo(dT)18 primer. Amplification of ERβ cDNA was carried out in a DNA thermal cycler at 94, 59, and 72 ^{o}C for 30 sec, 1 min, and 1 min, respectively, for 39 cycles using 50 pmol of each ERβ primer. Oligonucleotide primers were chosen from homologous parts of the coding region of the human ERβ gene. The sense primer for human ERβ was 5'-TAGTGGTCCATCGCCAGTTAT-3', and the antisense primer was 5'-GGGAGCACACTTCACCAT-3'. Amplification of the constitutive glyceraldehyde-3-phosphate dehydrogenase (GAPDH) cDNA was performed as an internal control for cDNA quantity and integrity, using 10 pmol of each primer of GAPDH, for 28 cycles at 94, 55, and 72 ^{o}C for 30 sec, 1 min, and 1 min, respectively. The primers of GADPH were: 5'-ATCACCATCTTCCAGGAGCG-3' for the sense primer and 5'-CCTGCTTCACCACCTTCTTG-3' for the antisense primer. The PCR fragments were separated using agarose gel electrophoresis and stained with ethidium bromide.

Results

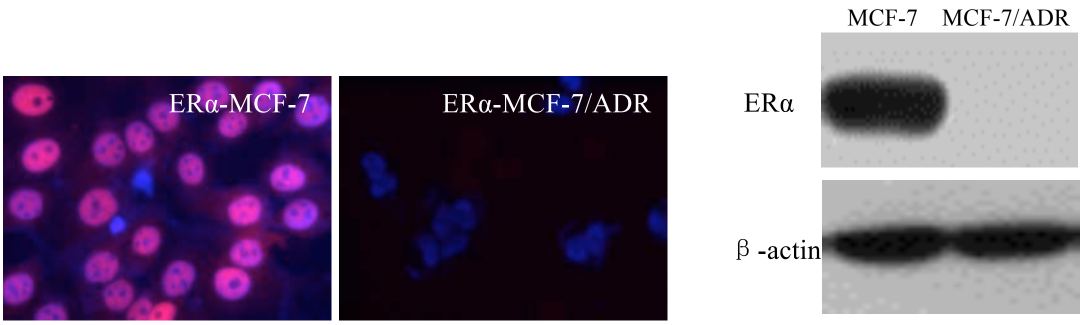

Supplemental Material, Figure 1. ERα status in MCF-7 and MCF-7/ADR cells was measured by immunofluorescence and Western Blot.

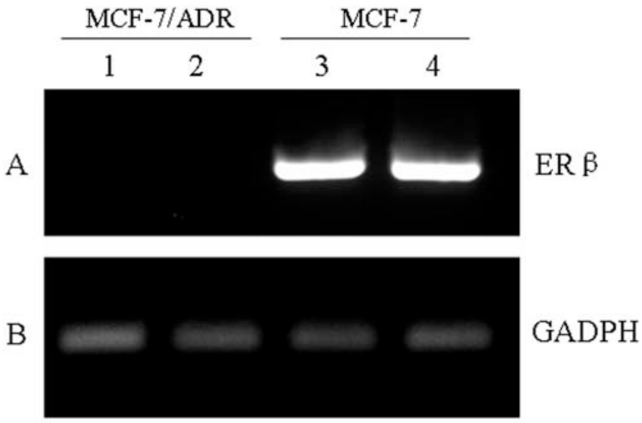

Supplemental Material, Figure 2. Detection of ERβ expression by using RT-PCR in MCF-7 and MCF-7/ADR cells. Line 1 and 2, RNA from MCF-7/ADR cells; Line 3 and 4, RNA from MCF-7 cells.
